# Clinical measurement of cellular DNA damage hypersensitivity in patients with DNA repair defects

**DOI:** 10.1186/s13023-022-02199-8

**Published:** 2022-02-14

**Authors:** Ola Hammarsten, Anna Lyytikäinen, Sofia Thunström, Torben Ek, Anders Fasth, Olov Ekwall, Sara Cajander, Emilie Wahren Borgström, C. I. Edvard Smith, Pegah Johansson

**Affiliations:** 1 Laboratory of Clinical Chemistry, Sahlgrenska University Hospital, Gothenburg, Sweden; 2 Department of Laboratory Medicine, Sahlgrenska Academy at the University of Gothenburg, Gothenburg, Sweden; 3 Department of Clinical Genetics, Sahlgrenska University Hospital, Gothenburg, Sweden; 4 Department of Internal Medicine and Clinical Nutrition, Sahlgrenska Academy, Gothenburg University, Gothenburg, Sweden; 5 Children’s Cancer Centre, Queen Silvia Children’s Hospital, Gothenburg, Sweden; 6 Department of Pediatrics, , Institution of Clinical Sciences, Sahlgrenska Academy, University of Gothenburg, Gothenburg, Sweden; 7 Department of Rheumatology and Inflammation Research, Gothenburg University, Gothenburg, Sweden; 8 Department of Infectious Diseases, Faculty of Medicine and Health, Örebro University, Örebro, Sweden; 9 Department of Infectious Diseases, The Immunodeficiency Unit, Karolinska University Hospital, Huddinge, Stockholm, Sweden; 10 Division of Infectious Diseases, Department of Medicine, Karolinska Institutet, Solna, Stockholm, Sweden; 11 Department of Laboratory Medicine, Karolinska Institutet, Huddinge, Sweden

**Keywords:** Cell division assay (CDA), γ-H2AX, DNA repair deficiency disorders, Ionizing radiation sensitivity, Mitomycin C sensitivity, Clinical diagnosis

## Abstract

**Background:**

DNA repair deficiency disorders are rare inherited diseases arising from pathogenic (disease-causing) variants in genes involved in DNA repair. There are no standardized diagnostic assays for the investigation of pathological significance of unknown variants in DNA repair genes. We hypothesized that our assays for measuring in vitro patient blood cell hypersensitivity to DNA-damaging agents can be used to establish the pathological significance of unknown variants in DNA repair genes. Six patients with variants in the DNA repair genes *PRKDC* (two siblings), *DCLRE1C* (two siblings), *NBN*, and *MSH6* were included. Here, we used the cell division assay (CDA) and the γ-H2AX assay, which were both developed and clinically validated by us, to measure patient cell hypersensitivity in response to ionizing radiation, mitomycin C, cytarabine and doxorubicin.

**Results:**

Radiation hypersensitivity was detected in the two patients with variants in the *PRKDC* gene (*p* < 0.0001 for both at 3.5 Gy), and the two patients with *DCLRE1C variants* (*p* < 0.0001 at 3.5 Gy for sibling 1 and *p* < 0.0001 at 1 Gy for sibling 2). The cells from the patients with the *PRKDC* variant were also deficient in removing γ-H2AX (*p* < 0.001). The cells from the patient with variants in the *NBN* gene were hypersensitive to mitomycin C (*p* = 0.0008) and deficient in both induction and removal of γ-H2AX in response to radiation.

**Conclusions:**

The combination of the CDA and the γ-H2AX assay is useful in investigating the significance of unknown variants in some DNA repair genes.

**Supplementary Information:**

The online version contains supplementary material available at 10.1186/s13023-022-02199-8.

## Background

DNA repair deficiency disorders are rare monogenic diseases caused by variants in the genes coding for proteins involved in DNA damage response and repair. These diseases may share some clinical features, such as growth retardation, neurological defects, premature ageing, skin alterations, telangiectasia, and xerosis cutis [1]. Variants in the same DNA repair gene can lead to highly diverse clinical outcomes. The variable clinical phenotypes, which often overlap with other diseases [2], and the lack of clinical assays to measure functional DNA repair deficiency, pose a challenge to the diagnosis of patients with unknown variants in the DNA repair genes.

As some patients with DNA repair defects are sensitive to specific types of DNA damage, patient cell sensitivity to different DNA-damaging agents may be a way to examine the functional deficiency of unknown variants in the DNA repair gene. For example, ataxia telangiectasia (AT) patients are sensitive to ionizing radiation (IR) [3], while fanconi anemia (FA) patients are sensitive to DNA interstrand crosslinking agents (ICLs), such as cyclophosphamide and mitomycin C [4]. In some cases, a heterogeneous pattern of sensitivity to some of the DNA-damaging therapies has been reported. For example, while all FA patients are sensitive to ICL therapy, only a subset of patients have shown radiation sensitivity [5].

There are no clinically standardized methods for measuring a patient’s cell sensitivity to DNA-damaging agents. The gold standard has been the clonogenic survival assay, carried out on fibroblast cultures or lymphoblastoid cell lines established from patient cells [6]. However, due to poor reproducibility and an analysis time of up to a few months, this assay is not suitable for clinical applications. We have therefore developed the cell division assay (CDA) to measure the relative sensitivity of patient cells to DNA-damaging agents in vitro [6]. The assay was optimized to correlate with the clonogenic survival assay [6], thereby serving as a surrogate for clonogenic cell survival. In contrast to the clonogenic assay, the CDA can be used to evaluate peripheral blood T cell sensitivity, and it is cheaper, faster and has greater precision. We have validated the assay using patients with variants of known clinical significance [6, 7].

Detection of H2AX phosphorylation (γ-H2AX) at the DNA double-strand break (DSB) sites has been shown to correlate with the number of DSBs and is used to monitor the cell response to agents that induce DSBs [8] such as IR [9, 10]. We have also optimized the flow cytometry γ-H2AX assay for clinical applications to measure the induction and repair of DSBs in patient cells in response to IR [11–14].

Here, we present the use of the CDA and γ-H2AX assay, as a useful combination to detect the pathological effect of unknown variants in the DSB repair pathway genes. Notably, many patients with DNA repair deficiency disorders also have an elevated risk of malignancies, and/or need for hematopoietic stem cell transplantation, and risk severe side effects of DNA-damaging therapies [9]. Therefore, knowledge of the patient’s sensitivity is not only useful for diagnostic purposes, but also essential before the possible treatment of patients.

## Results

### T cell sensitivity in patients with variants in the *PRKDC* gene

**Fig. 1:**
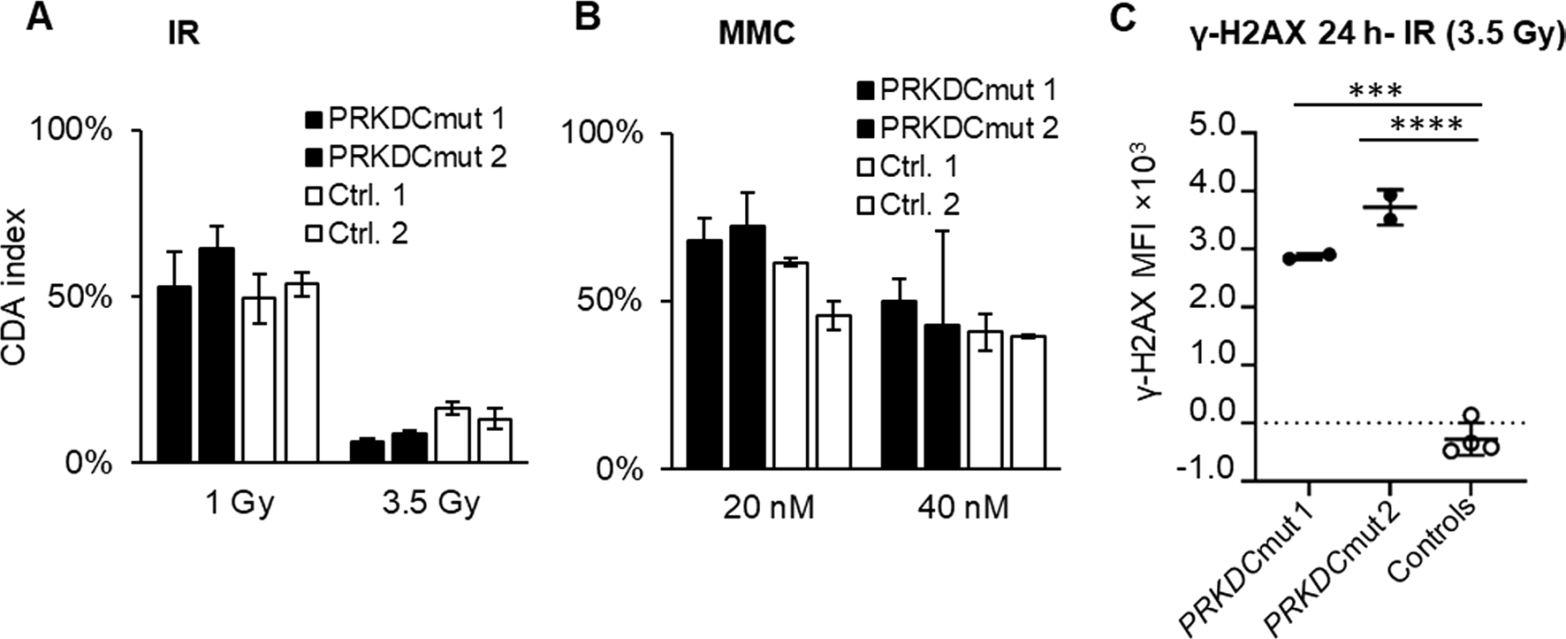
In vitro sensitivity of blood T cells from two siblings with variants in the *PRKDC* gene and two heathy controls using the CDA treated with the indicated doses of **A** IR or **B** MMC. The controls were assigned arbitrary numbers. Error bars indicate the standard deviation of technical replicates. **C** PBMC from patients and controls were treated with two doses of IR and the γ-H2AX MFI in the treated sample was corrected for the background by subtracting the γ-H2AX MFI in the non-treated samples at 24 h and plotted. Error bars indicate the standard deviation of the mean. The patient γ-H2AX MFI was compared with the controls using a two-tailed t test

Peripheral blood mononuclear cells (PBMC) from siblings with the same genetic variants in the *PRKDC* gene (Additional file 1: Table S1) were treated with IR and MMC in parallel and investigated with the CDA and γ-H2AX assays. When compared to the controls, the cells from the patients showed a two-fold increased sensitivity to the higher dose of IR (Fig. 1A). The cells from both patients were significantly more sensitive to IR compared with a large number of controls run over several days (*p* values in Table 1) (Additional file 1: Fig. S1A). On the other hand, patient cells had similar sensitivity to MMC treatment as the controls (Fig. 1B, Table 1 and Additional file 1: Fig. S1B). For both patients, the mean T cell proliferation in the untreated samples was lower than the average for the controls (*p* values in Table 1) (Additional file 1: Fig. S1A).

**Table 1:**
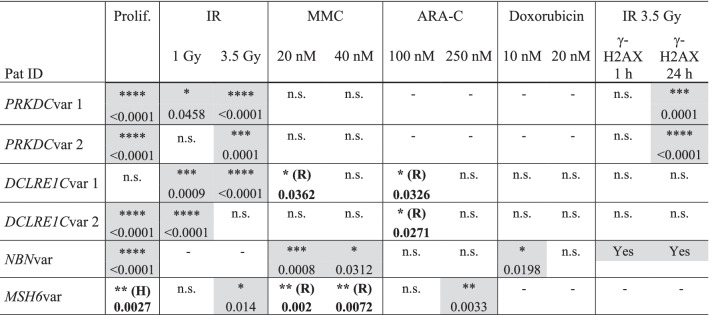
Summary of patient-cell sensitivity to different agents using the CDA and γ-H2AX assays

Significance and *p*-values were obtained from statistical analyses of patient samples relative to controls as shown in the figures and Additional file 1: Fig. S1

The table cells shaded in grey indicate significant hypersensitivity relative to the controls

(H): Higher proliferation rate than controls

(R): Resistance against agent relative to the controls

Induction of DSBs and their repair was also monitored in patient PBMC treated with IR, using our flow cytometry γ-H2AX assay [12]. The γ-H2AX induction was similar in patient and control samples (data not shown); however, the residual γ-H2AX after 24 h was significantly higher in patient cells than in the controls (Fig. 1C).

### T cell sensitivity in patients with variants in the *DCLRE1C* gene

**Fig. 2:**
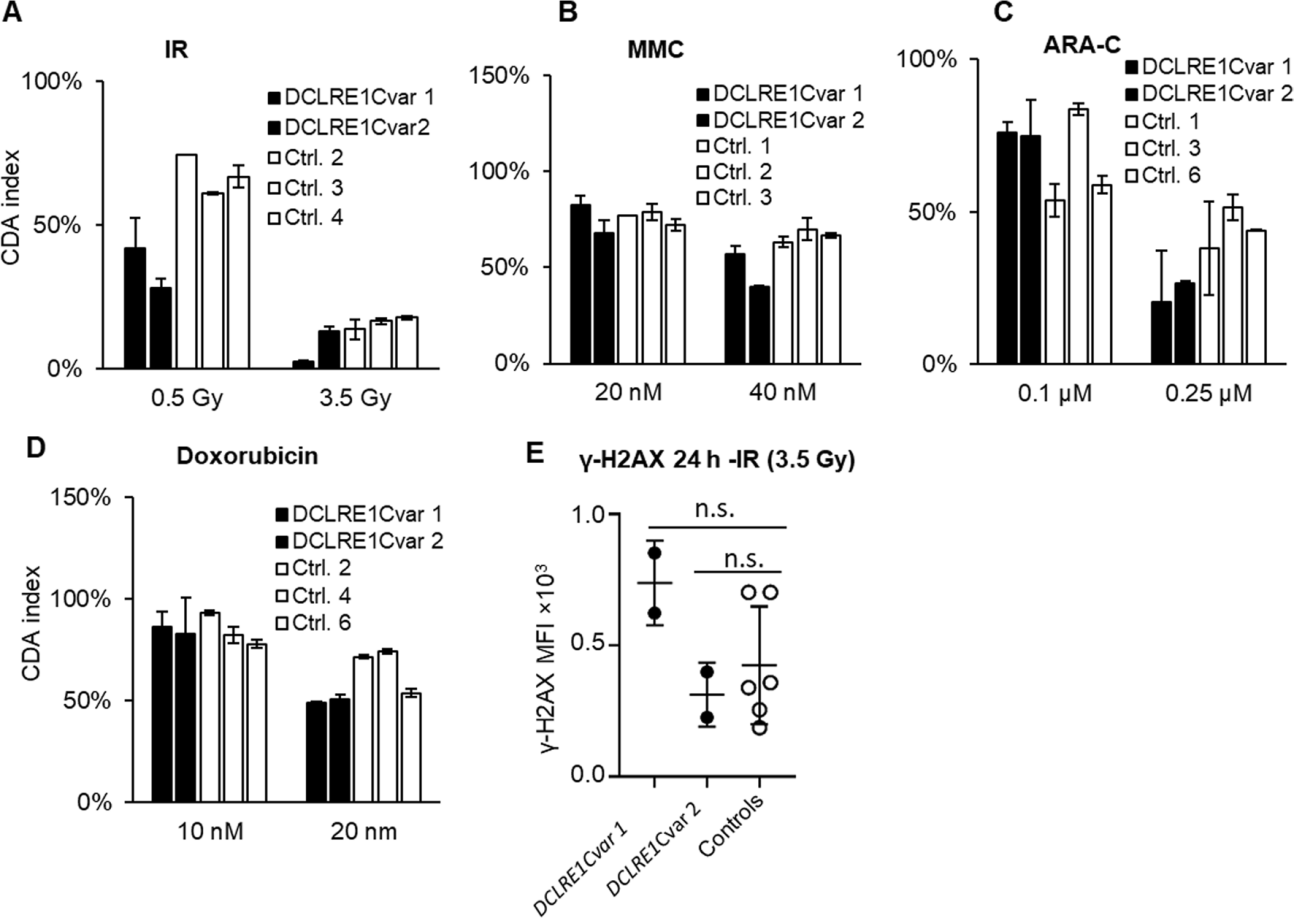
In vitro sensitivity of blood T cells from two siblings with variants in the *DCLRE1C* gene and heathy controls using the CDA treated with the indicated doses of **A** IR, **B** MMC, **C** ARA-**C**, and **D** doxorubicin. The controls were assigned arbitrary numbers. Error bars indicate the standard deviation of technical replicates. **E** PBMC from patients and controls were treated with two doses of IR and the γ-H2AX MFI in the treated sample was corrected for the background by subtracting the γ-H2AX MFI in the non-treated samples at 24 h and plotted. Error bars indicate the standard deviation of the mean. The patient γ-H2AX MFI was compared with the controls using a two-tailed t test

PBMC from two siblings with the same variants in the *DCLRE1C* gene (Additional file 1: Table S1) were analyzed as described above. The cells from both patients were more sensitive to IR at the higher dose, and for one of them also at the lower dose, relative to the controls (Fig. 2A). The cells from both patients were statistically more sensitive to IR at both doses when compared with a larger number of controls (*p* values in Table 1) (Additional file 1: Fig. S1A). Patient cells did not show hypersensitivity to MMC, doxorubicin, or cytarabine (ARA-C) (Fig. 2B-C, and Additional file 1: figure B-C). The CDA response to MMC and ARA-C was statistically higher than for controls (*p* values in Table 1), but the difference was very small. T cell proliferation in the untreated sample from one of the patients (*DCLRE1C*var 1) was comparable to the controls, while a severe and significant defect in proliferation was observed for *DCLRE1C*var 2 (*p* value in Table 1) (Additional file 1: Fig. S1F). Induction of DSBs and repair in response to IR was similar to the controls using the γ-H2AX assay (Fig. 2C).

### T cell sensitivity in patients with variants in the *NBN* gene

**Fig. 3:**
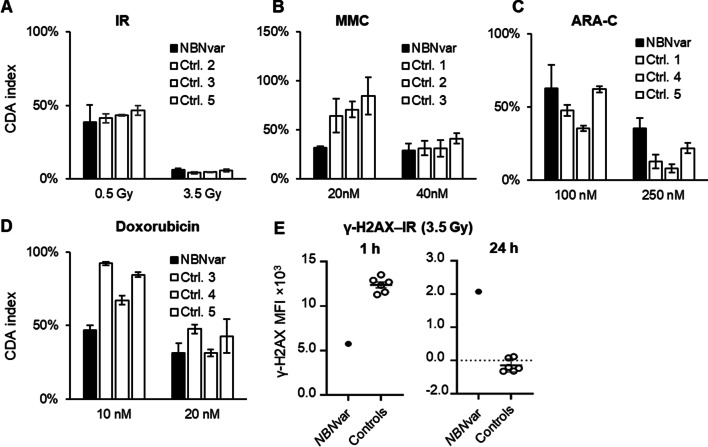
In vitro sensitivity of blood T cells from a patient with homozygous variants in the *NBN* gene and heathy controls using the CDA treated with the indicated doses of **A** IR, **B** MMC, **C** ARA-C, and **D** Doxorubicin. The controls were assigned arbitrary numbers. Error bars indicate the standard deviation of technical replicates. **E.** PBMC from patients and controls were treated with two doses of IR and the γ-H2AX MFI in the treated sample was corrected for the background by subtracting the γ-H2AX MFI in the non-treated samples at 1 h and 24 h and plotted. Error bars indicate the standard deviation of the mean. Due to the lack of material, the analysis was carried out once for the patient, but 5 × 10^3^ and 15 × 10^3^ cells were analyzed at 1 and 24 h, respectively

PBMC from a patient with homozygous pathogenic variants in the *NBN* gene (Additional file 1: Table S1) were analysed as described above. Surprisingly, the T cells from this patient were not hypersensitive to IR relative to the controls using the CDA (Fig. 3A). Since the CDA index for the controls run on the same day was not within the reference range (see Materials and Methods), the data from the patient could not be compared with the larger number of controls. The CDA indicated significant patient cell hypersensitivity to MMC (*p* values in Table 1) (Fig. 3B, and Additional file 1: Fig. S1B). The proliferation of untreated T cells from this patient was less than 5% relative to an average of approx. 90% for controls (*p* value in Table 1) (Additional file 1: Fig. S1F).

The γ-H2AX induction in IR-treated patient cells at 1 h was two-fold lower than the controls, and approximately 20 times higher than the controls at 24 h, indicating deficient DSB sensing and repair (Fig. 3F).

### T cell sensitivity in a patient with variants in a mismatch repair gene

**Fig. 4:**
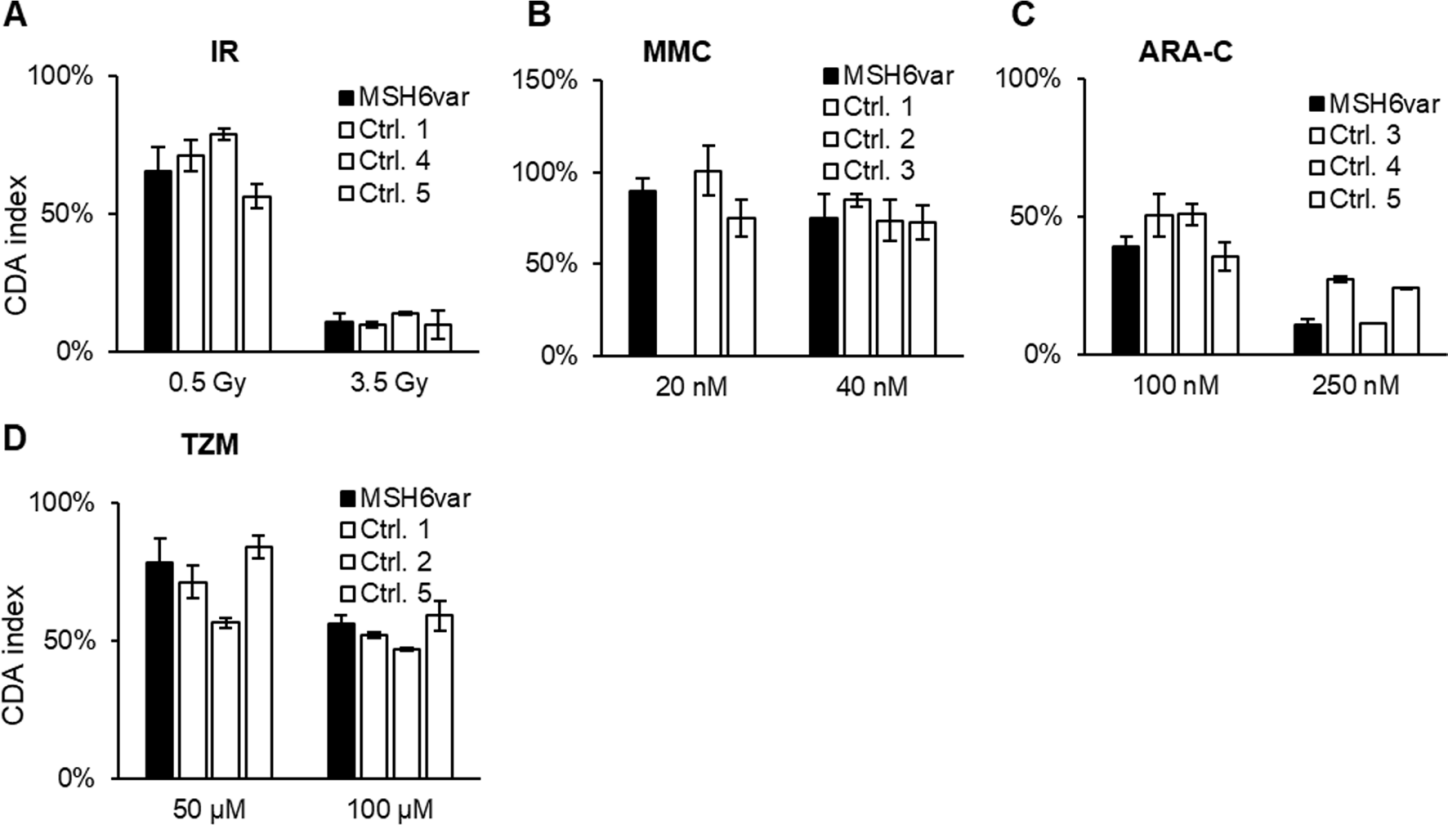
In vitro sensitivity of blood T cells from a patient with variants in the *MSH6 *gene and heathy controls using the CDA treated with the indicated doses of **A** IR, **B** MMC, **C** ARA-C, and **D** TZM. The controls were assigned arbitrary numbers. Error bars indicate the standard deviation of technical replicates

A patient with pathogenic variants in the *MSH6* gene (Additional file 1: Table S1) was sampled and analyzed for hypersensitivity to several DNA-damaging agents. The patient’s cells appeared to have a similar response to IR and MMC relative to the controls, but an increased sensitivity to ARA-C at the higher dose (Fig. 4A-C). When compared to a larger number of controls, the patient was slightly more sensitive to IR and ARA-C at the higher dose (*p* values in Table 1) (Additional file 1: Fig. S1A and C). The sensitivity of the patient’s cells to the methylating agent temozolomide (TZM) was also similar to the controls (Fig. 4D). The patient had a significantly higher rate of T cell proliferation (*p* value in Table 1) (Additional file 1: Fig. S1E).

## Discussion

Here, we used two functional assays to investigate the in vitro response of cells from different patients harboring variants in DNA repair genes to different DNA-damaging agents. Pathologic (disease-causing) variants in these genes are very rare and lead to heterogeneous and overlapping phenotypes [9]. Overall, our data showed differences in the patients’ cell response relative to the control individuals. Five of the patients investigated here carried variants in the DSB repair genes. These included genes coding for DNA-PKcs (*PRKDC* gene), Artemis (*DCLRE1C* gene) and Nibrin (*NBN* gene) proteins. Cells deficient in DSB repair proteins show increased sensitivity to IR [15]. The DSB repair pathways are also involved in V(D)J recombination, which is required for an efficient immune response. Therefore, defects in these pathways can also lead to severe combined immunodeficiency (SCID) with absence of T and B cells (T-B-SCID). Due to the radiosensitive phenotype, the patients are also referred to as RS-SCID [9, 10, 16]. Consistently, the cells from the patients with DSB repair defects analyzed here showed different aberrations in IR response, though the pattern of deficiency varied between the different patients according to the CDA and the γ-H2AX assays. This is probably due to the differences in the role of each protein in the DSB repair pathways [17–21]. DNA-PKcs is the catalytic subunit of DNA-PK, which is involved in the non-homologous end joining (NHEJ) DSB repair pathway. DNA-PK is responsible for sensing the DSB and phosphorylation of downstream effector proteins [17]. The samples from these patients were hypersensitive to IR according to the CDA and deficient in resolving γ-H2AX in agreement with a previous report [10]. Artemis is a nuclease, which is also involved in the NHEJ pathway of DSB repair and is recruited and phosphorylated by DNA-PK [18]. Samples from patients with Artemis deficiency were also hypersensitive to IR using the CDA but were normal in the γ-H2AX response in contrast to the DNA-PK deficient cells, indicating the γ-H2AX assay is not useful in detecting Artemis deficiency. Further, the level of IR hypersensitivity varied, even for siblings with the same genetic variation, suggesting that additional factors such as modifying genes, epigenetic and environmental factors may affect the severity of the phenotype. Damage induced by treatment with the DNA-crosslinking agent MMC involves the homologous recombination (HR) DSB repair pathway rather than the NHEJ. CDA using MMC treatment revealed hypersensitivity of cells from the patient with homozygous pathological variants in the *NBN* gene. This is consistent with Nibrin’s function of sensing DSBs and initiating the HR-DSB repair pathway, and a previous report of MMC sensitivity in Nibrin-deficient cells [20]. The cells from patients with variants in genes involving the NHEJ-DSB repair pathway (*PRKDC* and *DCLRE1C*) were not hypersensitive to MMC. Thus, our data indicate that the CDA specifically detects the hypersensitivity of patient cells and using panels of DNA-damaging agents can add to the functional investigation of these variants.

The CDA uses the activation and expansion of both CD4^+^ and CD8^+^ T cells, polyclonal T cells and antigen-specific T cells present in the PBMC. In line with the immunodeficiency of the patients, T cell activation/proliferation in untreated cells from four of the patients was significantly lower than among controls, again with varying levels. The deficiency was particularly severe in cells from the patient with the variants in the *NBN* gene.

Patients with defects in DSB repair and immunodeficiency often require hematopoietic stem cell transplantation (HSCT) and have an increased risk of malignancies [9, 19]. Therefore, it is critical to identify if the patients have increased sensitivity to other chemotherapeutic agents that could impact their preparation for HSCT or treatment for their malignancy. The CDA allows easy integration of multiple DNA-damaging agents in parallel, as demonstrated here.

A patient with pathological variants in the *MSH6* gene involved in the DNA mismatch repair (MMR) pathway was included, due to the very high predisposition of the patient to malignancy, and the risk of exposure to DNA-damaging therapies [22]. Notably, there are no reports indicating cells deficient in MMR exhibiting any treatment sensitivity. Analyses of sensitivity to additional DNA-damaging therapies suggested increased sensitivity of patient cells to a few agents and in some cases resistance relative to controls. The reason for the increased patient cell sensitivity to these agents is not known. This may be due to the genetic defect in *MSH*6, or other genetic variants in the lymphocytes, since the patient is prone to hypermutation. This sensitivity needs to be considered and investigated further if the patient is to be treated with similar agents. As cells with mutations in the MMR repair pathway are more resistant to apoptosis induced by O^6^-methylating agents, the methylating agent TZM was included for this patient. No difference in sensitivity to TZM was detected, but an abnormally high rate of T cell proliferation was observed, which may be due to aberrant cell cycle regulation in the cells from this patient. Notably, a significant resistance to MMC treatment was observed for cells from this patient, which has been reported for cells deficient in other MMR factors [23]. This further supports the ability of the CDA to specifically measure cell sensitivity, and detect individual variation.

One challenge for the validation of these assays for DNA repair deficiency disorders is that they are rare diseases. As the number of tested individuals increases, it is likely that the assay limitations and the reference range for a normal population response to different treatments will be more precisely determined. Another challenge is that our assays depend on the availability of normal blood, but since only a very small volume is required, this should be feasible in most labs with a hematology routine.

## Conclusion

Here, we present the use of the clinically validated CDA and γ-H2AX assay, as a useful combination in detecting the pathological effect of variants in genes coding for the DSB repair pathway proteins. These assays do not require skin biopsies or B cells, which are sometimes not present in these patients. Additionally, the assays are fast and more precise than the clonogenic survival assay, and allow evaluation of T cell activation in response to antigen stimulation. The relative ease of the CDA and γ-H2AX assay, the feasibility of integration in diagnostic routine analyses, and the short time frame [6] also allow individualized screening of the sensitivity pattern of patients to multiple agents prior to treatment. Finally, the assays have the potential to predict treatment sensitivity for cancer patients without a previous history of genetic defects, as there is a large individual variation in treatment-related side effects. This application is currently under investigation.

## Materials and methods

Patient samples: Six patients with variants in the DNA repair genes *PRKDC* (two siblings), *DCLRE1C* (two siblings), *NBN*, and *MSH6* were included. A brief description of the patients, their genetic variants and phenotype is given in Additional file 1: Table S1. Blood was taken in EDTA tubes from the patients. All blood samples were processed within 12 h.

Excess blood (EDTA tubes) with normal blood counts from routine hematology at the Sahlgrenska University Hospital central laboratory were used as controls. The controls used for different experiments were different individuals and were assigned an arbitrary number starting at 1 for each experiment. For the CDA assay a range of 31–35 controls were included for different treatments and doses. The number of controls for each treatment is indicated on the x axis in Additional file 1: Fig. S1. The controls were randomly selected, included both sexes and their age ranged from 18 to 75 years old.

Drug treatment: Stock solutions of cytarabine (ARA-C) (100 mM) and temozolomide (TZM) (Sigma-Aldrich) (50 mM) were prepared in dimethyl sulfoxide (DMSO) (Sigma-Aldrich) and stored in aliquots at −80 °C. Doxorubicin (Teva Sweden AB) (3.4 mM) was stored in aliquots at −80 °C. Mitomycin C (MMC) (12 mM) (Sigma-Aldrich) was prepared in DMSO and stored at 4 °C for a maximum of 3 months. Drugs were serially diluted in culture medium and added to the cell suspension at the indicated concentrations. The in vitro radiation was carried out in an RS 3400 Rad Source X-ray Blood Irradiator (Rad Source Technologies, GA, USA), using lead shielding to decrease the dose to the experimental doses indicated.

Cell division assay (CDA): CDA was carried out as previously described [8, 9]. The treatment of patient cells was carried out in duplicates or triplicates. The data analysis was carried out using the BD Accuri C6 software as previously described [8]. The T cell proliferation rate in the untreated sample was calculated as the percentage of EdU-positive cells in that sample using the BD Accuri C6 software.

Measurement of γ-H2AX by flow cytometry: H2AX phosphorylation was measured by flow cytometry analysis as previously described [12]. The data analysis was carried out using the BD Accuri C6 software as previously described (12). The γ-H2AX mean fluorescence intensity (MFI) for each time point was calculated by subtracting the γ-H2AX MFI of the non-treated sample harvested at the same time point.

Statistical analysis: Averages from technical replicates from each individual were plotted and the error bars indicate the standard deviation of the mean. The CDA index for each patient was plotted together with controls run on the same day. Where the controls were within the reference range for each treatment, as determined by at least 30 normal controls, the CDA index for the patient sample was statistically tested against the larger number of controls, carried out over several days, and presented graphically in Additional file 1: Fig. S1. Statistical analyses were carried out using the GraphPad prism 9.1.0 software (GraphPad Software). For the CDA, the replicate values were compared with the values obtained for controls (of different ages assayed on several occasions) using a two-tailed nonparametric Mann–Whitney test. The *p* values are outlined in Table 1.

## Supplementary Information

**Additional file 1.** **Table S1.** Patients included in the study. **Fig. S1.** In vitro sensitivity of blood T cells from patients compared to heathy controls using the CDA treated with indicated doses of; A. IR, B. MMC, C. Ara-C, and D. Doxorubicin. E. The proliferation rate of untreated T cells for each patient and 33 control individuals. Patient proliferation was assayed as four to six replicates. The number of controls is indicated for each dose and ranged from 18 to 75 years old. The line and error bars indicate the mean and standard deviation of the mean. Distribution of controls was not normal, and the patients were compared to the controls using the nonparametric two-tailed Mann-Whitney test.

## Data Availability

The datasets used and/or analyzed in the current study are available from the corresponding author on reasonable request.

## Abbreviations

AT: Ataxia telangiectasia
CDA: Cell division assay
ARA-C: Cytarabine
DMSO: Dimethyl sulfoxide
DSB: DNA double-strand break
FA: Fanconi anemia
HSCT: Hematopoietic stem cell transplantation
HR: Homologous recombination
ICLs: Interstrand crosslinking agents
IR: Ionizing radiation
MMR: Mismatch repair
MMC: Mitomycin C
NHEJ: Non-homologous end joining
PBMC: Peripheral blood mononuclear cells
RS-SCID: Radiosensitive SCID
T-B-SCID: SCID with absence of T and B cells
SCID: Severe combined immunodeficiency
TZM: Temozolomide
