# Supplementary material for: Clinical measurement of cellular DNA damage hypersensitivity in patients with DNA repair defects
Source: Orphanet J Rare Dis. 2022 Feb 14;17:50. doi: 10.1186/s13023-022-02199-8 (PMC8842932; doi:10.1186/s13023-022-02199-8)
Supplement: Supplementary file 1 — Additional file 1. Table S1. Patients included in the study. Fig. S1. In vitro sensitivity of blood T cells from patients compared to heathy controls using the CDA treated with indicated doses of; A. IR, B. MMC, C. Ara-C, and D. Doxorubicin. E. The proliferation rate of untreated T cells for each patient and 33 control individuals. Patient proliferation was assayed as four to six replicates. The number of controls is indicated for each dose and ranged from 18 to 75 years old. The line and error bars indicate the mean and standard deviation of the mean. Distribution of controls was not normal, and the patients were compared to the controls using the nonparametric two-tailed Mann-Whitney test. [file 13023_2022_2199_MOESM1_ESM.pdf]

Supplementary Table 1. Patients included in the study

**Pat ID/gene variant**	**Diagnosis**	**Variant description**	**Phenotype**
*PRKDC*var 1	CID	compound heterozygous variants (an in frame deletion and a missense variant)	Mild combined immunodeficiency presenting with, IgA deficiency, recurrent infections, vitiligo and atypical intestinal disease resembling celiac.
*PRKDC*var 2	CID		
*DCLRE1C*var 1	SCID	compound heterozygous variants (a large deletion and a missense variant)	Immunodeficiency, no B cells, recurrent respiratory tract infections and emphysema/bronchiectasis
*DCLRE1C*var 2	SCID		
*NBN*var	NBS	homozygous truncating variant	Microcephaly, growth retardation and developmental delay. An immunological defect affecting mainly the T cells, and recurrent infections.
*MSH6*var	CMMRD	compound heterozygous truncating variants	Atypical neurofibromatosis and multiple intracerebral abnormalities as well as multiple polyps and adenomas in the colon.

Abbreviations:		
	CID	Combined immunodeficiency
	SCID	Severe combined immunodeficiency
	NBS	Nijmegen breakage syndrome
	CMMRD	Constitutional Mismatch Repair Deficiency Syndrome

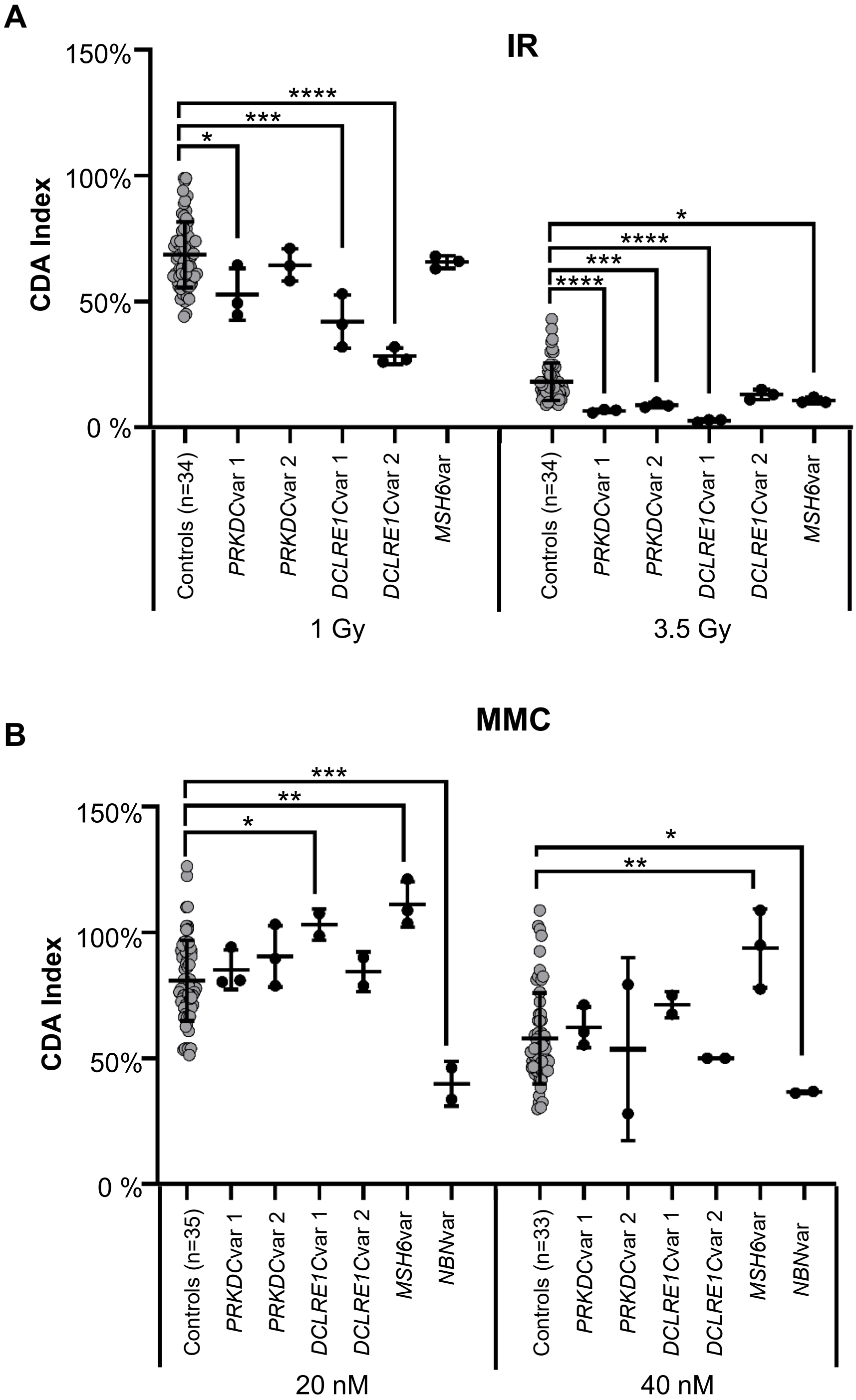
A
IR
150%
100%
50%
0 %
CDA Index
****
***
*
****
***
****
*
Controls (n=34)
PRKDCvar 1
PRKDCvar 2
DCLRE1Cvar 1
DCLRE1Cvar 2
MSH6var
Controls (n=34)
PRKDCvar 1
PRKDCvar 2
DCLRE1Cvar 1
DCLRE1Cvar 2
MSH6var
1 Gy
3.5 Gy
B
MMC
***
**
*
*
**
150%
100%
50%
0 %
CDA Index
Controls (n=35)
PRKDCvar 1
PRKDCvar 2
DCLRE1Cvar 1
DCLRE1Cvar 2
MSH6var
NBNvar
Controls (n=33)
PRKDCvar 1
PRKDCvar 2
DCLRE1Cvar 1
DCLRE1Cvar 2
MSH6var
NBNvar
20 nM
40 nM

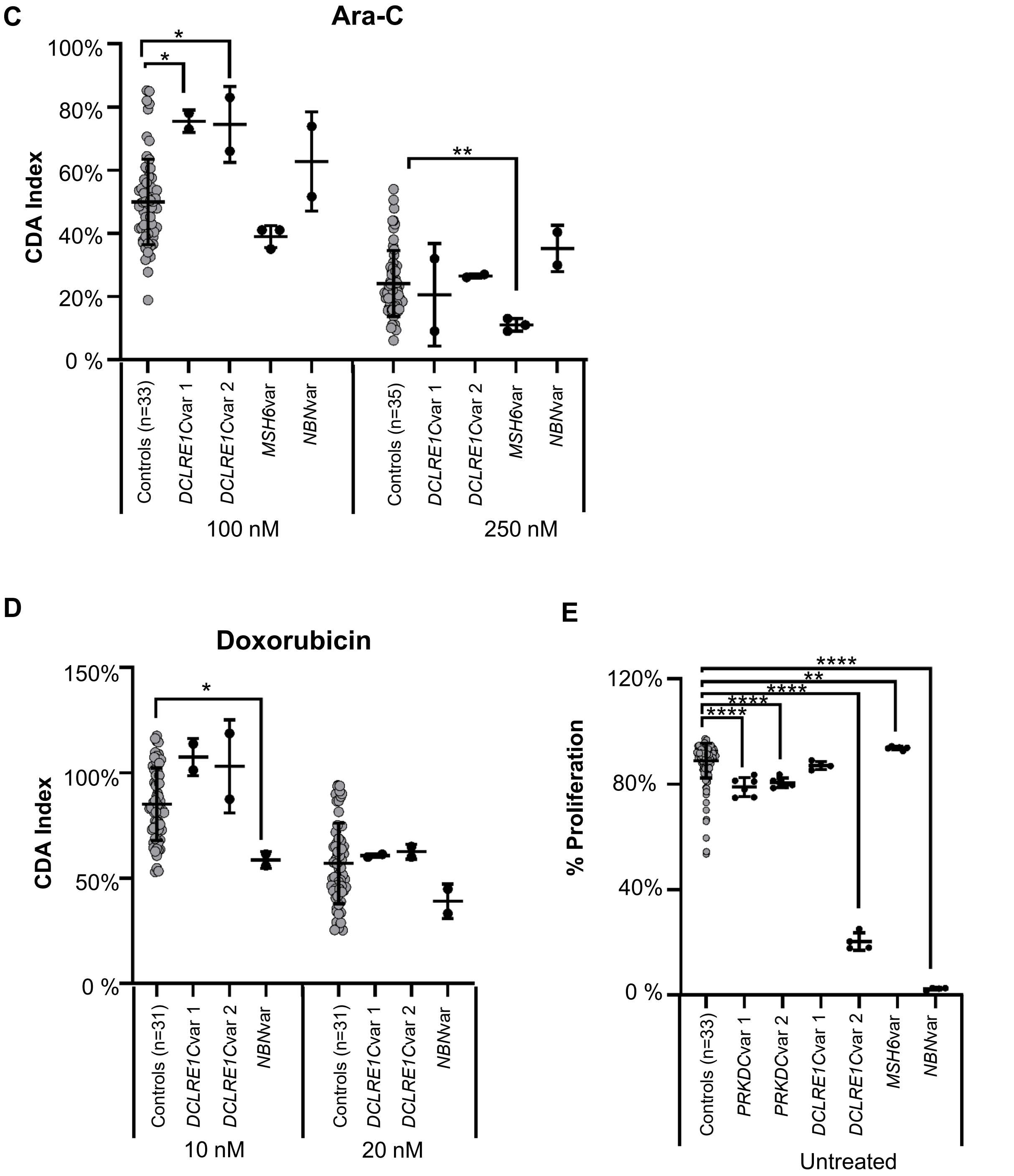

Supplmentary Figure 1. *In vitro* sensitivity of blood T cells from patients compared to heathy controls using the CDA treated with indicated doses of; **A.** IR, **B.** MMC, **C.** Ara-C, and **D.** Doxorubicin. **E.** The proliferation rate of untreated T cells for each patient and 33 control individuals. Patient proliferation was assayed as four to six replicates. The number of controls is indicated for each dose and ranged from 18 to 75 years old. The line and error bars indicate the mean and standard deviation of the mean. Distribution of controls was not normal, and the patients were compared to the controls using the nonparametric two-tailed Mann-Whitney test.
